# Tuberculosos pandemic and dissemination of drug resistant strains: a challenge for Bulgaria

**Published:** 2011-01-10

**Authors:** Violeta Valcheva, Igor Mokrousov, Olga Narvskaya, Nalin Rastogi, Nadya Markova

**Affiliations:** 1 Department of Infectious Diseases, The Stephan Angeloff Institute of Microbiology, Bulgarian Academy of Sciences, Sofia 1113, Bulgaria; 2 Laboratory of Molecular Microbiology, St. Petersburg Pasteur Institute, St. Petersburg, Russia; 3 Unité de la Tuberculose et des Mycobactéries, Institut Pasteur de Guadeloupe, Guadeloupe

## Background

Since early Neolithic, Europe as a whole and Balkans in particular were at the crossroads of human migrations thereby transmitting human pathogens across the continent. Bulgaria located near the Europe-Asia border was in the front of these migrations that left their imprint on the population structure of human pathogens circulating therein. A re-emergence and wide dissemination of multidrug-resistant tuberculosis (MDR-TB) threatens national control problems. The early detection of resistance to first line anti-TB drugs is essential for the efficient treatment and constitutes one of the priorities of TB control of MDR strains. The rate of the MDR-TB among newly diagnosed TB patients in Bulgaria was estimated to be 10.7% that is much higher than in the neighboring countries. Here we evaluated fast molecular methods to detect drug resistant TB and studied the distribution of resistant properties in different clonal lineages of *M. tuberculosis* in Bulgaria versus its neighbors.

## Methods

Drug-resistant and susceptible *M. tuberculosis* strains from newly–diagnosed patients were studied by different typing methods (spoligo-, IS6110-RFLP and 24-loci MIRU-VNTR typing). Mutations in the major gene targets related to drug resistance (*rpoB* RRDR*, katG315, inhA -15, embB306*) were detected by PCR and microarrays.

## Results

The population of *M. tuberculosis* in Bulgaria was found sufficiently heterogenous (24-VNTR based HGI=0.89). Mutation in *rpoB531* was detected in the remarkably high rate among RIF-resistant strains (65%). Mutations in *katG315* and *inhA* -15 were detected only in 50% of INH-resistant strains. The *embB306* mutation was found in 63% of EMB-resistant strains. Comparison with genotyping results did not identify any strain cluster linked to drug resistance.

## Conclusion

*M. tuberculosis* population in Bulgaria features several global, Balkan- and Bulgaria- specific lineages. *rpoB* RRDR and *embB306* mutations may serve for rapid genotypic detection of the majority of RIF and EMB-resistant *M. tuberculosis* strains in Bulgaria. The results for INH resistance are complex and more genes should be studied. The very high rate of *rpoB* S531L mutation may correlate with some specific features of the national TB control program (quality of the drug used) or is hypothetically linked to another molecular mechanism of RIF resistance. A local circulation of the particular clones appears to be an important factor to take into consideration in the molecular epidemiological studies of tuberculosis in Bulgaria. Emergence and spread of drug-resistant and MDR-TB in Bulgaria are not associated with any particular spoligotype or MIRU-VNTR genotype.

